# Supplementary material for: HIVprotI: an integrated web based platform for prediction and design of HIV proteins inhibitors
Source: J Cheminform. 2018 Mar 9;10:12. doi: 10.1186/s13321-018-0266-y (PMC5845081; doi:10.1186/s13321-018-0266-y)
Supplement: Supplementary file 2 — Additional file 2. Source code of HIVProtI web server. [file 13321_2018_266_MOESM2_ESM.zip › HIVprotI_Source-code/hivprotI/weka-3-6-6/doc/weka/core/SerializationHelper.html]

SerializationHelper


---


|  |  |  |
| --- | --- | --- |
| **Overview** \| **Package** \| **Class** \| **Tree** \| **Deprecated** \| **Index** \| **Help** \| **Weka's home** | |  |
| **PREV CLASS**   **NEXT CLASS** | **FRAMES**    **NO FRAMES**     **All Classes** |  |
| SUMMARY: NESTED \| FIELD \| CONSTR \| METHOD | DETAIL: FIELD \| CONSTR \| METHOD |  |


---


## weka.core Class SerializationHelper

```
java.lang.Object
  weka.core.SerializationHelper
```

**All Implemented Interfaces:**: RevisionHandler

---

``` public class SerializationHelper extends java.lang.Object implements RevisionHandler ```

A helper class for determining serialVersionUIDs and checking whether
classes contain one and/or need one. One can also serialize and deserialize
objects to and fro files or streams.

**Version:**
:   $Revision: 6229 $

**Author:**
:   fracpete (fracpete at waikato dot ac dot nz)

---

| **Field Summary** | |
| --- | --- |
| `static java.lang.String` | `SERIAL_VERSION_UID`             the field name of serialVersionUID. |


**Constructor Summary**

`SerializationHelper()`


| **Method Summary** | |
| --- | --- |
| `java.lang.String` | `getRevision()`             Returns the revision string. |
| `static long` | `getUID(java.lang.Class c)`             reads or creates the serialVersionUID for the given class. |
| `static long` | `getUID(java.lang.String classname)`             reads or creates the serialVersionUID for the given class. |
| `static boolean` | `hasUID(java.lang.Class c)`             checks whether the given class contains a serialVersionUID. |
| `static boolean` | `hasUID(java.lang.String classname)`             checks whether the given class contains a serialVersionUID. |
| `static boolean` | `isSerializable(java.lang.Class c)`             checks whether a class is serializable. |
| `static boolean` | `isSerializable(java.lang.String classname)`             checks whether a class is serializable. |
| `static void` | `main(java.lang.String[] args)`             Outputs information about a class on the commandline, takes class name as arguments. |
| `static boolean` | `needsUID(java.lang.Class c)`             checks whether a class needs to declare a serialVersionUID, i.e., it implements the java.io.Serializable interface but doesn't declare a serialVersionUID. |
| `static boolean` | `needsUID(java.lang.String classname)`             checks whether a class needs to declare a serialVersionUID, i.e., it implements the java.io.Serializable interface but doesn't declare a serialVersionUID. |
| `static java.lang.Object` | `read(java.io.InputStream stream)`             deserializes from the given stream and returns the object from it. |
| `static java.lang.Object` | `read(java.lang.String filename)`             deserializes the given file and returns the object from it. |
| `static java.lang.Object[]` | `readAll(java.io.InputStream stream)`             deserializes from the given stream and returns the object from it. |
| `static java.lang.Object[]` | `readAll(java.lang.String filename)`             deserializes the given file and returns the objects from it. |
| `static void` | `write(java.io.OutputStream stream, java.lang.Object o)`             serializes the given object to the specified stream. |
| `static void` | `write(java.lang.String filename, java.lang.Object o)`             serializes the given object to the specified file. |
| `static void` | `writeAll(java.io.OutputStream stream, java.lang.Object[] o)`             serializes the given objects to the specified stream. |
| `static void` | `writeAll(java.lang.String filename, java.lang.Object[] o)`             serializes the given objects to the specified file. |

**Methods inherited from class java.lang.Object**

`equals, getClass, hashCode, notify, notifyAll, toString, wait, wait, wait`

**Field Detail**

### SERIAL\_VERSION\_UID

```
public static final java.lang.String SERIAL_VERSION_UID
```

:   the field name of serialVersionUID.

    **See Also:**: Constant Field Values


**Constructor Detail**

### SerializationHelper

```
public SerializationHelper()
```


**Method Detail**

### isSerializable

```
public static boolean isSerializable(java.lang.String classname)
```

:   checks whether a class is serializable.

    :   **Parameters:**: `classname` - the class to check **Returns:**: true if the class or one of its ancestors implements the Serializable interface, otherwise false (also if the class cannot be loaded)

---


### isSerializable

```
public static boolean isSerializable(java.lang.Class c)
```

:   checks whether a class is serializable.

    :   **Parameters:**: `c` - the class to check **Returns:**: true if the class or one of its ancestors implements the Serializable interface, otherwise false

---


### hasUID

```
public static boolean hasUID(java.lang.String classname)
```

:   checks whether the given class contains a serialVersionUID.

    :   **Parameters:**: `classname` - the class to check **Returns:**: true if the class contains a serialVersionUID, otherwise false (also if the class is not implementing serializable or cannot be loaded)

---


### hasUID

```
public static boolean hasUID(java.lang.Class c)
```

:   checks whether the given class contains a serialVersionUID.

    :   **Parameters:**: `c` - the class to check **Returns:**: true if the class contains a serialVersionUID, otherwise false (also if the class is not implementing serializable)

---


### needsUID

```
public static boolean needsUID(java.lang.String classname)
```

:   checks whether a class needs to declare a serialVersionUID, i.e., it
    implements the java.io.Serializable interface but doesn't declare a
    serialVersionUID.

    :   **Parameters:**: `classname` - the class to check **Returns:**: true if the class needs to declare one, false otherwise (also if the class cannot be loaded!)

---


### needsUID

```
public static boolean needsUID(java.lang.Class c)
```

:   checks whether a class needs to declare a serialVersionUID, i.e., it
    implements the java.io.Serializable interface but doesn't declare a
    serialVersionUID.

    :   **Parameters:**: `c` - the class to check **Returns:**: true if the class needs to declare one, false otherwise

---


### getUID

```
public static long getUID(java.lang.String classname)
```

:   reads or creates the serialVersionUID for the given class.

    :   **Parameters:**: `classname` - the class to get the serialVersionUID for **Returns:**: the UID, 0L for non-serializable classes (or if the class cannot be loaded)

---


### getUID

```
public static long getUID(java.lang.Class c)
```

:   reads or creates the serialVersionUID for the given class.

    :   **Parameters:**: `c` - the class to get the serialVersionUID for **Returns:**: the UID, 0L for non-serializable classes

---


### write

```
public static void write(java.lang.String filename,
                         java.lang.Object o)
                  throws java.lang.Exception
```

:   serializes the given object to the specified file.

    :   **Parameters:**: `filename` - the file to write the object to: `o` - the object to serialize **Throws:**: `java.lang.Exception` - if serialization fails

---


### write

```
public static void write(java.io.OutputStream stream,
                         java.lang.Object o)
                  throws java.lang.Exception
```

:   serializes the given object to the specified stream.

    :   **Parameters:**: `stream` - the stream to write the object to: `o` - the object to serialize **Throws:**: `java.lang.Exception` - if serialization fails

---


### writeAll

```
public static void writeAll(java.lang.String filename,
                            java.lang.Object[] o)
                     throws java.lang.Exception
```

:   serializes the given objects to the specified file.

    :   **Parameters:**: `filename` - the file to write the object to: `o` - the objects to serialize **Throws:**: `java.lang.Exception` - if serialization fails

---


### writeAll

```
public static void writeAll(java.io.OutputStream stream,
                            java.lang.Object[] o)
                     throws java.lang.Exception
```

:   serializes the given objects to the specified stream.

    :   **Parameters:**: `stream` - the stream to write the object to: `o` - the objects to serialize **Throws:**: `java.lang.Exception` - if serialization fails

---


### read

```
public static java.lang.Object read(java.lang.String filename)
                             throws java.lang.Exception
```

:   deserializes the given file and returns the object from it.

    :   **Parameters:**: `filename` - the file to deserialize from **Returns:**: the deserialized object **Throws:**: `java.lang.Exception` - if deserialization fails

---


### read

```
public static java.lang.Object read(java.io.InputStream stream)
                             throws java.lang.Exception
```

:   deserializes from the given stream and returns the object from it.

    :   **Parameters:**: `stream` - the stream to deserialize from **Returns:**: the deserialized object **Throws:**: `java.lang.Exception` - if deserialization fails

---


### readAll

```
public static java.lang.Object[] readAll(java.lang.String filename)
                                  throws java.lang.Exception
```

:   deserializes the given file and returns the objects from it.

    :   **Parameters:**: `filename` - the file to deserialize from **Returns:**: the deserialized objects **Throws:**: `java.lang.Exception` - if deserialization fails

---


### readAll

```
public static java.lang.Object[] readAll(java.io.InputStream stream)
                                  throws java.lang.Exception
```

:   deserializes from the given stream and returns the object from it.

    :   **Parameters:**: `stream` - the stream to deserialize from **Returns:**: the deserialized object **Throws:**: `java.lang.Exception` - if deserialization fails

---


### getRevision

```
public java.lang.String getRevision()
```

:   Returns the revision string.

    :   **Specified by:**: `getRevision` in interface `RevisionHandler`
    :   **Returns:**: the revision

---


### main

```
public static void main(java.lang.String[] args)
                 throws java.lang.Exception
```

:   Outputs information about a class on the commandline, takes class
    name as arguments.

    :   **Parameters:**: `args` - the classnames to check **Throws:**: `java.lang.Exception` - if something goes wrong


---


|  |  |  |
| --- | --- | --- |
| **Overview** \| **Package** \| **Class** \| **Tree** \| **Deprecated** \| **Index** \| **Help** \| **Weka's home** | |  |
| **PREV CLASS**   **NEXT CLASS** | **FRAMES**    **NO FRAMES**     **All Classes** |  |
| SUMMARY: NESTED \| FIELD \| CONSTR \| METHOD | DETAIL: FIELD \| CONSTR \| METHOD |  |


---
